# Morphological study of tooth development in podoplanin-deficient mice

**DOI:** 10.1371/journal.pone.0171912

**Published:** 2017-02-21

**Authors:** Kenyo Takara, Naoki Maruo, Kyoko Oka, Chiaki Kaji, Yuji Hatakeyama, Naruhiko Sawa, Yukinari Kato, Junro Yamashita, Hiroshi Kojima, Yoshihiko Sawa

**Affiliations:** 1 Department of Oral Growth & Development, Fukuoka Dental College, Fukuoka, Japan; 2 Department of Odontology, Fukuoka Dental College, Fukuoka, Japan; 3 Department of Morphological Biology, Fukuoka Dental College, Fukuoka, Japan; 4 Department of Oral and Maxillofacial Surgery, Fukuoka Dental College, Fukuoka, Japan; 5 Department of Regional Innovation, Tohoku University Graduate School of Medicine, Sendai, Japan; Kyungpook National University School of Medicine, REPUBLIC OF KOREA

## Abstract

Podoplanin is a mucin-type highly *O*-glycosylated glycoprotein identified in several somatyic cells: podocytes, alveolar epithelial cells, lymphatic endothelial cells, lymph node stromal fibroblastic reticular cells, osteocytes, odontoblasts, mesothelial cells, glia cells, and others. It has been reported that podoplanin-RhoA interaction induces cytoskeleton relaxation and cell process stretching in fibroblastic cells and osteocytes, and that podoplanin plays a critical role in type I alveolar cell differentiation. It appears that podoplanin plays a number of different roles in contributing to cell functioning and growth by signaling. However, little is known about the functions of podoplanin in the somatic cells of the adult organism because an absence of podoplanin is lethal at birth by the respiratory failure. In this report, we investigated the tooth germ development in podoplanin-knockout mice, and the dentin formation in podoplanin-conditional knockout mice having neural crest-derived cells with deficiency in podoplanin by the *Wnt1* promoter and enhancer-driven Cre recombinase: *Wnt1-Cre;Pdpn*^*Δ/Δ*^mice. In the *Wnt1-Cre;Pdpn*^*Δ/Δ*^mice, the tooth and alveolar bone showed no morphological abnormalities and grow normally, indicating that podoplanin is not critical in the development of the tooth and bone.

## Introduction

Podoplanin is a mucin-type highly *O*-glycosylated [1] glycoprotein identified in the rat kidney glomerular epithelial cell (podocyte) foot processes [2]. Podoplanin is highly negative charged by a high sialic acid content [1–6]. Mouse 38-kDa and human 36-kDa podoplanin cloned from lymphoid tissue have been termed gp38 [7] and gp36 [8] respectively. Podoplanin is commonly known as a useful lymphatic endothelial cell marker because blood endothelial cells lack podoplanin. Podoplanin null mice have defects in the lymphatic pattern formation with pronounced lymphedema at birth and show blood-filled lymphatics [9–11]. Kato et al. have reported details of podoplanin bindings to the platelet transmembrane protein CLEC-2 causing platelet agglutination by the binding activity [6]. It has been reported that the platelet agglutination on the lymphatic endothelium plays a key role in the sprouting of two jugular lymph sacs from the junction of the subclavian veins with the anterior cardinals (future internal jugular vein)[11]. Further, it has been established that podoplanin interact with Rho GTPase for cell process stretching [12]. Podoplanin plays a key role in the lymph node expansion by signaling between lymph node stromal fibroblastic reticular cells and CLEC-2 delivering dendritic cells [13]. The CLEC-2 binding rapidly causes podoplanin uncoupling from RhoA and permits cytoskeleton relaxation in reticular cells, and cell process stretching [13].

Podoplanin was first detected on the cell surface of osteocytes, osteoblasts, and odontoblasts in rodent bone and tooth [14–16]. Mouse podoplanin is identical to the partial cDNA OTS-8, which is the earliest description of the podoplanin gene cloned from mouse osteoblast-like MC3T3-E1 cells treated with 12-O-tetradecanoylphorbol-13-acetate [17], and it is identical to PA2.26 from mouse epidermal keratinocytes [3]. Rat podoplanin protein has been reported as RTI40 in alveolar epithelial cells [1] and E11 antigen from osteocytes [14]. In culture, primary cells of osteoblasts are E11 negative and start to express E11 with aging and mineralization [18]. The produced amounts of E11 are higher in MLO-Y4 osteocyte-like cells than in osteoblast cell lines and primary osteoblasts. Podoplanin cloned from rat type I alveolar cells have also been termed T1alpha [19,20]. The first reported podoplanin-deficient mice with the 129/SvEv background died at birth because the lungs cannot be inflated to the volumes allowing breath [21]. Podoplanin is also expressed in the choroid plexus [22,23], mesothelial cells [24,25], epidermal basal layer cells [9], tooth germ epithelial cells, salivary gland myoepithelium [26–30], thymus type I epithelial cells, prostate myofibroblasts, follicular dendritic cells, and immature cells such as fetal germ cells and developing Sertoli cells [31–34], in the normal nervous system and perineurium [35,36], and in nervous system tumors [37–40].

Overall, this suggests that podoplanin plays a number of different roles in contributing to cell functioning and growth by signaling. It appears likely that podoplanin plays a critical role in the development of the type I alveolar lung cells, and in the lymphatic circulation system and lymph node expansion in vivo, and that podoplanin-RhoA interaction induces cytoskeleton relaxation and the cell process stretching in fibroblastic cells, osteocytes, and other cell lines in vitro. However, the function of podoplanin in other podoplanin-positive organs, particularly in the hard tissue, has not been established as podoplanin absence is lethal to mice because of the occurrence of respiratory failure at birth as mice lines with podoplanin-floxed alleles have not yet. In *Wnt1-Cre* transgenic mice which express Cre recombinase under the control of the wingless-related MMTV integration site 1 (*Wnt1*) promoter and enhancer have been extensively used in the study of the neural crest derivatives [41]. Here, we report the tooth and alveolar bone development in podoplanin-conditional knockout mice in which podoplanin is absent in neural crest-derived cells: *Wnt1-cre* transgenic mice bred to mice having podoplanin-floxed alleles (*Pdpn*^fl/fl^).

## Materials and methods

The animal study was performed to achieve the project goal: a morphological investigation of conditionally podoplanin-deficient mice where alleles of podoplanin in the neural crest cells are inactivated. The studies here used C57BL/6N (wild type) mice, C57BL/6N mice with *Pdpn*^KO1st^ allele (*Pdpn*^*+/-*^, *Pdpn*^*-/-*^), C57BL/6JxCBA/J:*Wnt1-Cre* mice, C57BL/6N mice with floxed *Pdpn* allele (*Pdpn*^fl/fl^), and C57BL/6NJxCBA/J:*Wnt1-Cre*;*Pdpn* cKO mice with *Pdpn*^*Δ/+*^, *Pdpn*^*Δ/Δ*^ allele in *Wnt1* expressing cells) with six mice in each group (2/cage). The manuscript was prepared following the ARRIVE guidelines.

### Animals

The experimental protocol for animal use was reviewed and approved by the Animal Experiment Committee of Fukuoka Dental College in accordance with the principles of the Helsinki Declaration. Breeding and experiments were performed in a room with a 100% controlled atmosphere which had passed an examination for bacteria and is located in the Fukuoka Dental College Animal Center. Mice grew normally and lived healthily under conventional atmosphere conditions with normal feeding in cages and rooms in which the temperature (22°C) and humidity (55%) were completely controlled. The mice were housed with an inverse 12 hour day-night cycle with lights on from 7:00pm.

Humane endpoints were used in the experiments as a rapid and accurate method for assessing the health status of the mice, that is, mice with lost ability to ambulate (inability to access food or water) were euthanized by induction anesthesia (1 l/min of 2% isoflurane mixed with 30% oxygen and 70% nitrous oxide with an anaesthetic apparatus) followed by cervical dislocation and intraperitoneal injections with 3.5% chloral hydrate (10 ml/kg, trichloroacetaldehyde monohydrate, Kanto Chemical, Tokyo, Japan) in the saline.

### Generation of knockout first

**Fig 1:**
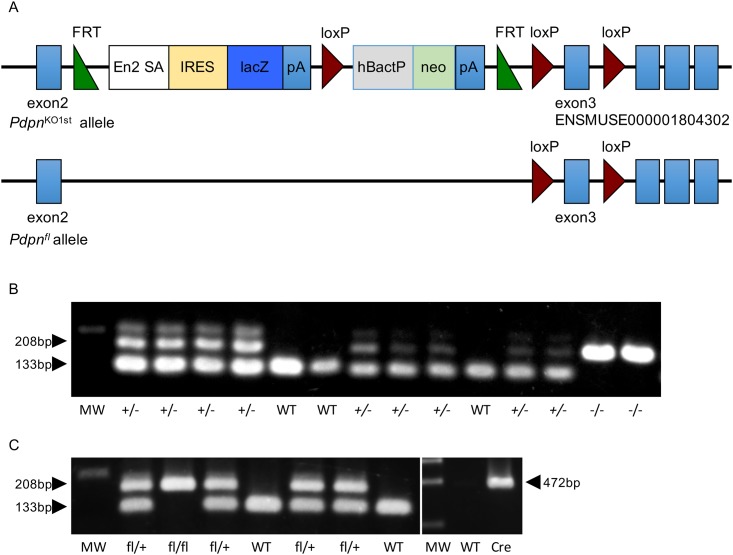
**(A) *Pdpn***^**KO1st**^ **allele with promoter-driven cassette and *Pdpn***^**fl**^ **allele.** ES cells (genetic background, C57BL/6N-A^tm1Brd^) having knockout-first allele by an insertion of a promoter-driven cassette (HTGR03003_Z_2_G05) in podoplanin gene located in Chr4 (143267431-143299564bp) was used. The cassette flanked by L1L2 gateway sites (L1L2_Bact_P) is inserted at position 143274509 of chromosome 4 upstream of exon of podoplanin located in Chr4 (143267431–143299564 bp). The cassette is composed of a short flippase recombination enzyme (Flp)-recognition target (FRT), reporter, and a Cre recombinase recognition target (loxP). Cre-loxP system from bacteriophage P1 is analogous to Flp-FRT system from *Saccharomyces cerevisiae* and recombines a pair of target sequences. The first FRT site is followed by the reporter which is a reading frame-independent LacZ gene trap cassette: splice acceptor of mouse En2 exon 2 (En2-SA), the internal ribosome entry site from encephalomyocarditis virus (ECMV IRES), Escherichia coli lacZ gene encoding the reporter enzyme β-galactosidase (lacZ), and simian virus 40 polyadenylation signal (pA). The first loxP site is followed by the neomycin selection cassette which is composed of human beta-actin promoter (hBactP) driving the neomycin-resistance gene (selectable marker neomycin phosphotransferase, neo), pA, a second FRT site, and a second loxP site. A third loxP site is inserted downstream of the targeted exon (synthetic loxP region, 22973–23052) at position 143273615, therefore, *Pdpn* exon 3 is flanked by loxP sites. A reporter knockout mouse not crossed with flp deleter mouse was used as *Pdpn* KO^1st^ mouse. Breeding with flp recombinase-deleter mouse created mouse carrying a floxed allele by subsequent excision of the targeting cassette. This study did not use mice removed the βact-neo cassette and the critical exons, and carrying a lacZ tagged null allele by applying Cre recombinase to the original version of the allele. Subsequently breeding *Pdpn*-floxed mouse with *Wnt1-Cre* mouse created mouse carrying a *Pdpn* exon3 null allele in the *Wnt1*-expressng cells. Cre-Lox analogous to Flp-FRT recombination is a site-specific recombination system consists of a Cre recombinase that recombines a pair of short target sequences called the lox sequences. The Cre enzyme and the original lox site called the loxP sequence are derived from bacteriophage P1. **(B) Genotyping of *Pdpn***^**KO1st**^ **mice.** *Pdpn*^KO1st^ mice having one mutant allele (+/-) by insertion of the cassette shown in (A) show two bands (133 and 208bp) or more cross-reaction band of higher molecular weight than the two. *Pdpn*^KO1st^ mice having two mutant alleles (-/-) show one band (208bp) and mice without *Pdpn* mutation show one band (133bp). (C) Genotyping of *Pdpn*^fl^ mice and *Wnt1*-Cre mice. *Pdpn*^fl/+^ mice having one *Pdpn* exon3-floxed mutant allele (fl/+) show two bands (133 and 208bp). *Pdpn* ^fl/fl^ mice having two mutant alleles (fl/fl) show one band (208bp) and mice without *Pdpn* mutation show one band (133bp). The Cre band is observed in genes from mice with Cre (472bp).

The targeting vector of the podoplanin gene (*Pdpn*) was purchased from EUCOMM (European Conditional Mouse Mutagenesis Program) which allows reporter-tagging and conditional mutation of the gene-of-interest and the generation of knockout first was entrusted to Transgenic Inc. (Fukuoka, Japan): allele name, *Pdpn*^tm1a(EUCOMM)Wtsi^; genetic background, C57BL/6N-A^tm1Brd^ (Fig 1A). The targeting vector is the promoter-driven targeting cassette and consists of the gene-trap cassette followed by the selection cassette through the loxP site. The targeting vector has three loxP sites with the first and second loxP sites sandwiching the gene-trap cassette. The gene-trap cassette contains a splice acceptor (SA) and an internal ribosome entry site (IRES) upstream of a lacZ reporter gene followed by a polyadenylation (pA) signal. The IRES-lacZ trapping cassette is placed at 5′ of a loxP-flanked, promoter-driven, neomycin-resistance selection cassette, which lies immediately upstream of exon 3. The *lacZ* cassette is able to apply in the generation of reporter-tagged animals expressing lacZ in the tissue that expresses the gene-of-interest. The selection cassette consists of a neomycin resistance gene (NeoR) driven by an autonomous promoter (hBactP) and pA signal. When the targeting vector, HTGR03003_Z_2_G05, is successfully inserted in the *Pdpn* gene domain downstream of the promoter, the gene is inactivated, and LacZ expresses instead. At EUCOMM this gene-trap cassette is used as a consensus structure of the targeting vector. The targeted allele *Pdpn*^*gt*^, containing the gene trap and selection cassettes is a conventional knockout-first allele [42], as the insertion of the cassettes disrupts the targeted gene splicing in C57BL/6N embryonic stem (ES) cells [42]. The *Pdpn* gene knockout-first allele, also referred to as *Pdpn*^KO1st^, was made by the reporter-tagged insertion with conditional potential (Transgenic Inc., Fukuoka, Japan). The *Pdpn*^KO1st^ mice were generated from chimeric mice with the Pdpn-targeted ES cells in which the genetic background is C57BL/6NCrj. Although the cassettes should be removed before the investigation of the phenotype development in the *Pdpn*^KO1st^, it is thought that *Pdpn*^KO1st^ mice containing *Pdpn*^*gt*^ homozygously are useful because of the absence of anomalies in mice having *Pdpn*^*gt*^ heterozygously, and the insertion of the *cassettes* would not affect the gene around the *Pdpn*^*gt*^ allele in this case. The promoter-driven targeting cassette which consists of the gene-trap and selection cassettes is flanked by flippase (Flp) recognition target FRT sites; one the upstream of the IRES-lacZ cassette and one between the upstream of the neomycin-resistance cassette and the second loxP site before exon 3 in order to simultaneously remove both the gene trap IRES-*lacZ* and the neomycin-resistance cassettes. The removal of the targeting cassette by Flp generates a *Pdpn* conditional KO allele including loxP sites flanking exon 3 (floxed exon 3). The exon3 is common to all *Pdpn* transcript variants and is critical in podoplanin protein development. The exon3 deletion creates a frame-shift mutation in the *Pdpn* expression. A third *loxP* site is inserted immediately after the exon 3 to remove the exon 3 and generate the *Pdpn*-inactivated mice in which both alleles of *Pdpn* exon 3 are homozygous *null*. Mice carrying the *Pdpn*^*gt*^ allele heterozygously were first bred with mice carrying a ubiquitously expressed Flp, *ACTB*:*FLPe* (B6;SJL-Tg(ACTFLPe)9205Dym/J, JAX 003800), in order to remove the promoter-driven targeting cassette by the Flp-mediated recombination *in vivo*. This breeding left a single FRT site and floxed exon 3 behind, enabling a true conditional knock-out by the deletion of exon 3. This allele is referred to as *Pdpn*^*fl*^. Mice carrying both the homozygous *Pdpn*^*fl*^ alleles (*Pdpn*^*fl/fl*^) and the *Wnt1*-expressing tissue specific heterozygous *Cre* recombinase gene were generated to obtain mice for the *Wnt1*-expressing tissue specific deletion of *Pdpn* exon 3, using *Pdpn*^*fl/fl*^ mice and the *Cre* recombinase gene transgenic mice: B6.Cg-Tg(*Wnt1-Cre*)11Rth Tg(*Wnt1-GAL4*)11Rth/J (C57BL/6JxCBA/J:*Wnt1-Cre*, Jax 009107). Deletion of exon 3 causes a frame shift and starts a premature stop codon near the 5′ end of exon 4 or 5 depending on splicing variants, thereby disrupting translation of the *Pdpn*. This allele is referred to as *Pdpn*^*Δ*^. Deficiency of podoplanin by the *Wnt1* promoter and enhancer-driven Cre recombinase occurs in the neutral crest-derived tissue expressing *Wnt1-Cre* in the *Wnt1-Cre;Pdpn*^*Δ/Δ*^ mice. We examined the conditional mutant C57BL/6NJxCBA/J:*Wnt1-Cre;Pdpn*^*Δ/Δ*^ mice with the *Wnt1*-expressing tissue specific *Pdpn* deletion (*Wnt1-Cre;Pdpn*^*Δ/Δ*^), as well as the wild-type (*Pdpn* ^*+/+*^) and heterozygous (*Pdpn*^*Δ/+*^) littermate controls carrying the *Cre* transgene.

### Genotyping

Genomic DNA from tail was isolated with a QIAamp DNA Blood and Tissue Kit (Qiagen, Hilden, Germany). All procedures were performed according to protocols provided by the manufacturers, and, in all cases, the duration of each procedure was recorded. The PCR was performed by 30 cycles for amplification using the Ex Taq hot start version (Takara Bio Inc., Otsu, Japan) with 50 pM of primer sets: The *Pdpn* targeted allele with the whole trapping cassette (6.5kbp); Pdpn_sc5AF1 (forward) 5'-CAGTGAGATTCTATAGGGCTGC, LacZ_AS1 (reverse): 5'-TTGTAAAACGACGGGATCTTCC; *Pdpn* without loxP site (wild, 133bp) and *Pdpn* including the third loxP site (208bp)(synthetic loxP region, 22973–23052)(Fig 1B); loxS1 (forward) 5'-AGGAAGAATCCCACACCAGG, loxAS1 (reverse): 5'-TGTAGGGAGCTACCGCTAGG. The primer sets of loxS1 and loxAS1 were basically used for detecting mice having *Pdpn*^KO1st^ and *Pdpn*^*fl/fl*^ alleles (Fig 1B). The PCR products were separated on 2% agarose gel (NuSieve; FMC, Rockland, ME, USA) and visualized by Syber Green (Takara). The correct size of the amplified PCR products was confirmed by gel electrophoresis and the amplification of accurate targets was confirmed by sequence analysis. The Cre recombinase gene driven by *Wnt1* promoter/enhancer (KC845567) was detected by PCR products (472bp)(Fig 1C) using the primer sets: 5'-CGTTTTCTGAGCATACCTGGA (forward), 5'-ATTCTCCCACCGTCAGTACG (reverse).

### Subjects

The wild type mice, *Pdpn* knockout-first B6 mice on the 18.5th day of pregnancy, and 2 week *Wnt1-Cre;Pdpn*^*Δ/Δ*^ mice were used (n = 10). Mice were euthanized by induction anesthesia (1 l/min of 2% isoflurane mixed with 30% oxygen and 70% nitrous oxide with an anaesthetic apparatus) followed by cervical dislocation and intraperitoneal injections with 3.5% chloral hydrate (10 ml/kg, trichloroacetaldehyde monohydrate, Kanto Chemical, Tokyo, Japan) and sodium pentobarbital (10 ml/kg, Nembutal, Abbott Laboratories, North Chicago, IL, USA) in the saline. Maxillary tissue including the upper molar was obtained from the wild and knockout-first mice at embryonic day 18.5 (E18.5), and from the *Wnt1-Cre;Pdpn*^*Δ/Δ*^ after euthanasia.

### Immunohistochemistry

Kawamoto’s film method with tungsten carbide blade was used for the sectioning so that intact hard and soft tissue can be observed without decalcification [43]. After the subjects were embedded in super cryoembedding medium (Leica Microsystems Japan, Tokyo, Japan) and rapidly frozen using liquid N_2_, sagittal undecalcified frozen sections (4 μm) of tissue including the upper incisor region were cut in a cryostat (Leica Microsystems, Wetzlar, Germany) with tungsten carbide blade. The sections were fixed in 100% ethanol for 30 sec at RT and subsequently immersed in 100% methanol for 30 sec at -20°C, treated with 0.1% goat serum for 30 min at 20°C, and then treated for 8 hrs at 4°C with PBS containing 0.1% goat serum and the following primary antibodies (1 μg/ml): hamster monoclonal anti-mouse podoplanin (AngioBio Co., Del Mar, CA) and rabbit anti-nephrin (Abcam plc., Cambridge, UK). After the treatment with primary antibodies the sections were washed three times in PBS for 10 min and immunostained for 0.5 hr at 20°C with 0.1 μg/ml of the second antibodies: Alexa Fluor (AF) 488 or 568-conjugated goat anti-hamster IgG and anti-rabbit IgG (Probes Invitrogen Com., Eugene, OR). The immunostained sections were mounted in 50% polyvinylpyrrolidone solution and examined by fluorescence microscopy (BZ-8100, Keyence Corp., Osaka, Japan) or confocal laser-scanning microscopy (LSM710, Carl Zeiss, Jena, Germany) with an x63 oil Plan Apochromatic objective lens.

### Measurement of the area of immunostaining

The podoplanin-stained area was measured on the five different spots (0.36 mm x 0.36 mm) in the renal section images using ImageJ (National Institutes of Health, Bethesda, MD). The relative expression amounts of podoplanin were expressed by the mean of the ratio: podoplanin-positive area in lung lobes (x20 magnification) and alveoli (x200 magnification) / scanned area.

### Statistics

All experiments were carried out five times, repeatedly, and data are expressed as mean + SD. The statistical significant differences (*p*< 0.01) were determined by one-way ANOVA and the unpaired two-tailed Student’s *t* test with STATVIEW 4.51 software (Abacus concepts, Calabasas, CA, USA).

## Results

### Immunostaining of *Pdpn*^KO1st^ mice for podoplanin

**Fig 2:**
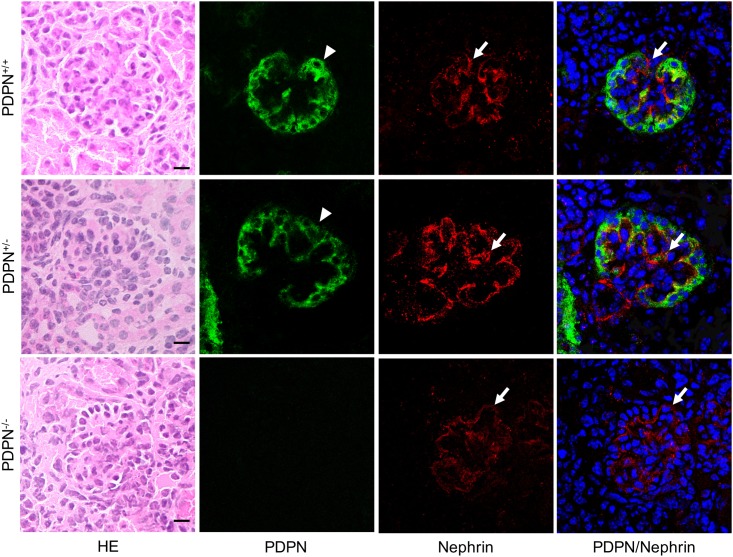
Immunostaining of the glomeruli in *Pdpn*^KO1st^ mice for podoplanin (PDPN) and nephrin. In the hematoxylin-eosin (HE) staining, there are no abnormalities in the glomeruli of the *Pdpn*^+/-^ and *Pdpn*^-/-^ mice. The expression of PDPN (arrowheads) is observed in the glomeruli of the wild type *Pdpn*^+/+^ and *Pdpn*^+/-^ mice, but not in the *Pdpn*^-/-^ mice. The expression of nephrin (arrows) is observed in the *Pdpn*^-/-^ and *Pdpn*^+/-^ mice, as well as in the *Pdpn*^+/+^ mice. The merged images show that there are double-positive podocytes and only nephrin-positive diaphragm area. Bar: 10μm.

In the kidney of *Pdpn*^KO1st^ mice, the expression of podoplanin was observed in the glomeruli of *Pdpn*^+/-^ mice as well as the wild type *Pdpn*^+/+^ mice, but not in the *Pdpn*^-/-^ mice (Fig 2). The expression of nephrin was observed in *Pdpn*^-/-^ and *Pdpn*^+/-^ mice, as well as in the wild type mice. It was observed that the podocytes express podoplanin and nephrin, and the area of the diaphragm between podocytes express only nephrin. There were no abnormalities in the development of glomeruli in the *Pdpn*^+/-^ and *Pdpn*^-/-^ mice.

**Fig 3:**
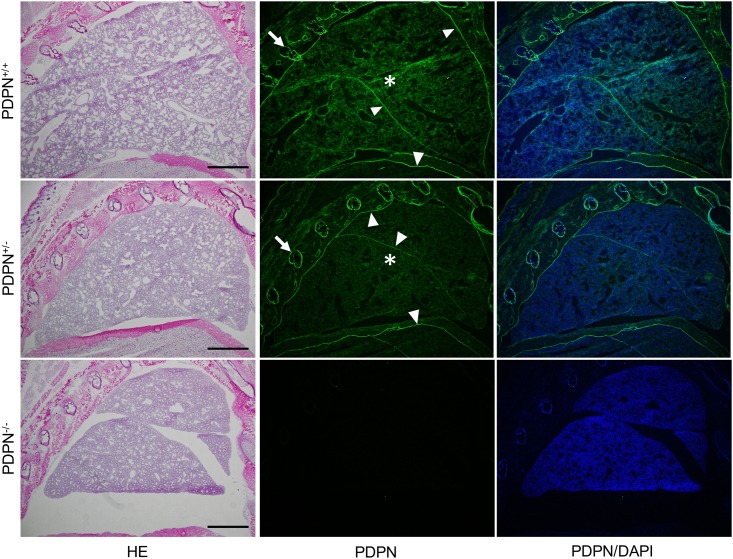
Immunostaining of the thoracic cage and lung parenchyma in *Pdpn*^KO1st^ mice for podoplanin (PDPN). In the hematoxylin-eosin (HE) staining, atrophy is observed in the *Pdpn*^-/-^ mice. The expression of podoplanin (arrowheads) is observed in the lung of the wild type *Pdpn*^+/+^ mice and *Pdpn*^+/-^ mice, but not in the *Pdpn*^-/-^. It is observed that the PDPN-positive areas are the mesothelia (arrowheads) of diaphragmatic pleura, costal pleura, and visceral pleura. It is also observed that costal bone (arrows) and lung parenchyma (asterisks) are PDPN positive. Staining densities of parenchyma PDPN are weaker in the *Pdpn*^+/-^ mice than in the *Pdpn*^+/+^ mice. Bar: 1 mm.

**Fig 4:**
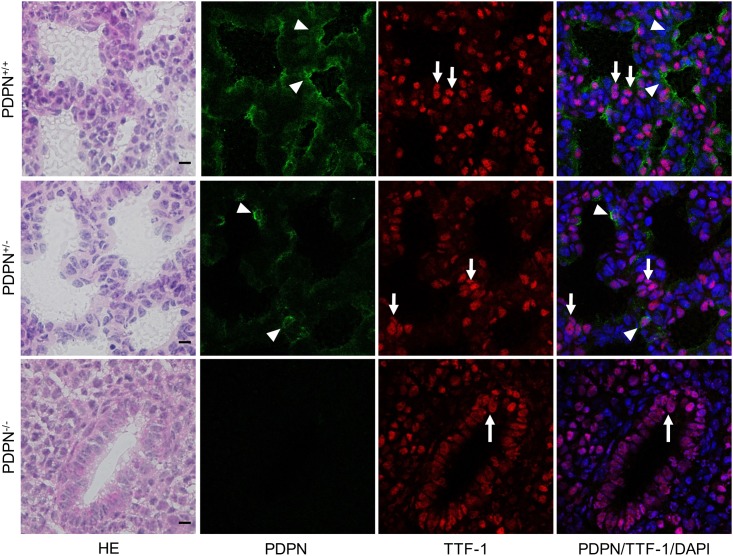
Immunostaining of the alveoli in the *Pdpn*^KO1st^ mice for podoplanin (PDPN) and thyroid transcription factor-1 (TTF-1). In the wild type *Pdpn*^+/+^ and *Pdpn*^+/-^ mice, there are the PDPN-positive type I alveolar epithelial cells among the TTF-1-positive type II alveolar epithelial cells. The PDPN expression area of the type I alveolar epithelial cells (arrowheads) is fewer in the *Pdpn*^+/-^ mice than in the *Pdpn*^+/+^ mice. In the *Pdpn*^-/-^ mice, the terminal ends of the respiratory tree consist of the alveolar ducts and respiratory bronchioles with only TTF-1-positive type II alveolar epithelial cells (arrows), lacking alveolar sacs with PDPN-positive type I alveolar epithelial cells. Bar: 10μm.

In the thoracic cage and lung of *Pdpn*^KO1st^ mice, there were no abnormalities in the development of pleura and costal bone in the *Pdpn*^+/-^ and *Pdpn*^-/-^ mice but atrophy of the lung was observed in the *Pdpn*^-/-^ mice (Fig 3). In the *Pdpn*^+/+^ and *Pdpn*^+/-^ mice, it was observed that the terminal ends of the respiratory tree, the pulmonary alveoli, consist of alveolar sacs and alveolar ducts whereas there were fewer developed intact alveoli in the *Pdpn*^+/-^ mice than in the *Pdpn*^+/+^mice, and there was disordered development in the *Pdpn*^-/-^ mice (S1 Fig). The expression of podoplanin was observed in the pleura mesothelial cells, costal bone, and lung parenchyma of *Pdpn*^+/-^ mice as well as in the *Pdpn*^+/+^ mice, but not observed in the *Pdpn*^-/-^ mice. The podoplanin expression of the alveoli in the *Pdpn*^+/-^ mice was weaker than in the *Pdpn*^+/+^ mice, but not observed in the *Pdpn*^-/-^ mice. In ImageJ analysis for the immunostained area, the podoplanin expression amounts on lung lobes and alveoli diaphragmatic pleura are significantly higher in the wild type *Pdpn*^+/+^ mice than in the *Pdpn*^+/-^ mice (S2 Fig). In the *Pdpn*^+/+^ and *Pdpn*^+/-^ mice, there were podoplanin-positive type I alveolar epithelial cells among the thyroid transcription factor-1 (TTF-1)-positive type II alveolar epithelial cells (Fig 4). The podoplanin-positive area of the type I alveolar epithelial cells in *Pdpn*^+/-^ mice were smaller than in the wild type *Pdpn*^+/+^ mice. In the *Pdpn*^-/-^ mice, the terminal ends of the respiratory tree consisted of the alveolar duct and respiratory bronchioles with only TTF-1-positive type II alveolar epithelial cells, but lacked alveolar sacs with podoplanin-positive type I alveolar epithelial cells.

**Fig 5:**
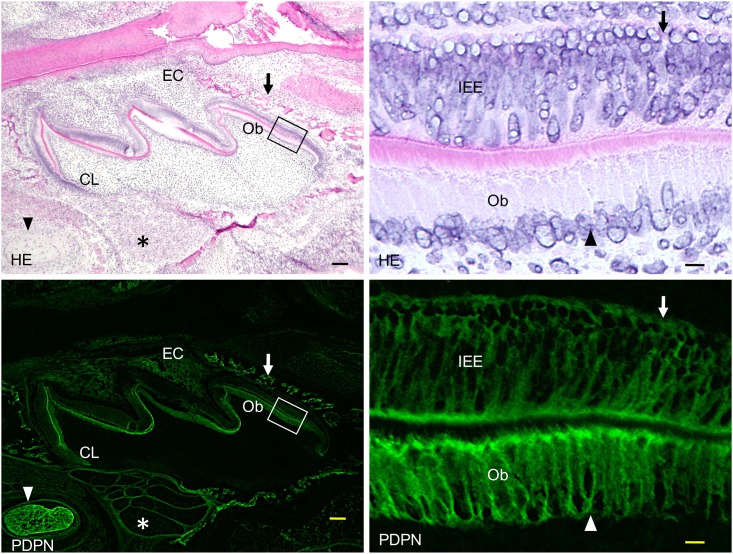
Immunostaining of a wild type *Pdpn*^+/+^ molar tooth germ at the early bell stage for podoplanin (PDPN). In the left images of hematoxylin-eosin staining (HE) and PDPN immunostaining, PDPN expression is observed in the enamel cord (EC), cervical loop (CL), and odontoblasts (Ob). Nerve sheaths (asterisk), Meckel’s cartilage (arrowhead), and bone (arrow) also exhibit a strong expression of podoplanin. In the right images at the higher magnification of the part highlighted by the box, PDPN expression is observed in the inner enamel epithelia (IEE, arrows) and odontoblasts (Ob, arrowheads). Bar: left 200μm, right 10μm.

**Fig 6:**
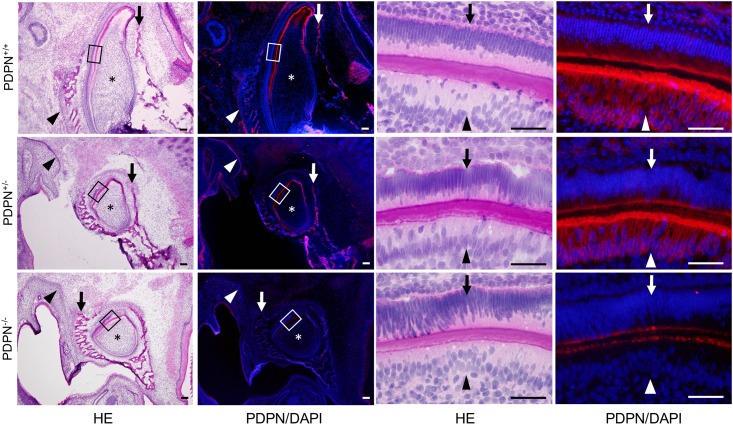
Immunostaining of a *Pdpn*^KO1st^ mouse lower incisor tooth germ sagittal section at the late bell stage for podoplanin (PDPN). In the hematoxylin-eosin (HE) staining, there are no abnormalities in the tooth germ with dentin matrix, bone, or nerve of the *Pdpn*^+/-^ and *Pdpn*^-/-^ mice. In the left immunostained images, the expression of PDPN is observed in the tooth germ (asterisks), in the alveolar bone (arrows), and in the sheath of the inferior alveolar nerve (arrowheads) of the wild type *Pdpn*^+/+^ mice, and in the *Pdpn*^+/-^ mice to a weaker extent than in the *Pdpn*^+/+^ mice. There is no immunostaining observed in the *Pdpn*^-/-^ tissue except for a non-specific reaction. In the right images at the higher magnification of the parts highlighted by the boxes in the left images, the expression of PDPN is observed in the odontoblasts (arrowheads), and in the inner enamel epithelial cells (arrows), of the wild type *Pdpn*^+/+^ mice, and of the *Pdpn*^+/-^ mice to a weaker extent than in the *Pdpn*^+/+^ mice. There is no immunostaining observed in the *Pdpn*^-/-^ tissue except for the cross reaction to the dentin matrix. Bar: 100μm.

In the mandible of wild type mice, the expression of podoplanin was observed in the tooth germ, nerve sheaths, and Meckel’s cartilage (Fig 5). In the tooth germ of the wild type mice, the expression of podoplanin was observed in the enamel cord, cervical loop, inner and outer enamel epithelia, and odontoblasts. In the mandible of *Pdpn*^KO1st^ mice, the expression of podoplanin was observed in the tooth germ, nerve sheaths, and alveolar bone in the *Pdpn*^+/-^ mice, whereas it was not observed in the *Pdpn*^-/-^ mice (Fig 6). The expression of podoplanin in the mandible was weaker in the *Pdpn*^+/-^ mice than in *Pdpn*^+/+^ mice. There seems to be no abnormalities in the development of tooth germs, bone, or nerves in the *Pdpn*^+/-^ and or *Pdpn*^-/-^ mice apparently (Fig 6).

### Immunostaining of *Wnt1-Cre;Pdpn* conditional knockout mouse tooth for podoplanin

**Fig 7:**
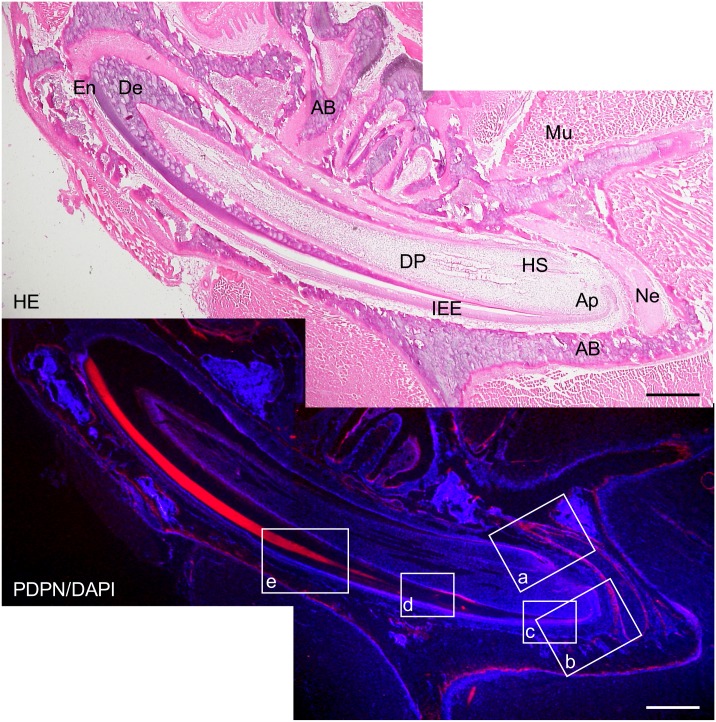
Immunostaining of a 2-week *Wnt1-Cre;Pdpn*^*Δ/+*^ mouse lower incisor sagittal section for podoplanin (PDPN). In the hematoxylin-eosin (HE) staining, there are no abnormalities in the bone, dentin or enamel formation. The expression of PDPN is observed in the Hertwig’s epithelial root sheath (HS), in the odontoblast layer at the edge of the dental pulp (DP), in the apical bud (Ap), in the inner enamel epithelial cells (IEE), in the periosteum at the edge of the alveolar bone (AB), and in the sheath of the inferior alveolar nerve (Ne). No expression of PDPN is observed in the dentin (De), dental pulp fibroblasts (DP), or in the muscle (Mu). There is the cross-reaction to the enamel of the labial side. Bar: 500μm.

**Fig 8:**
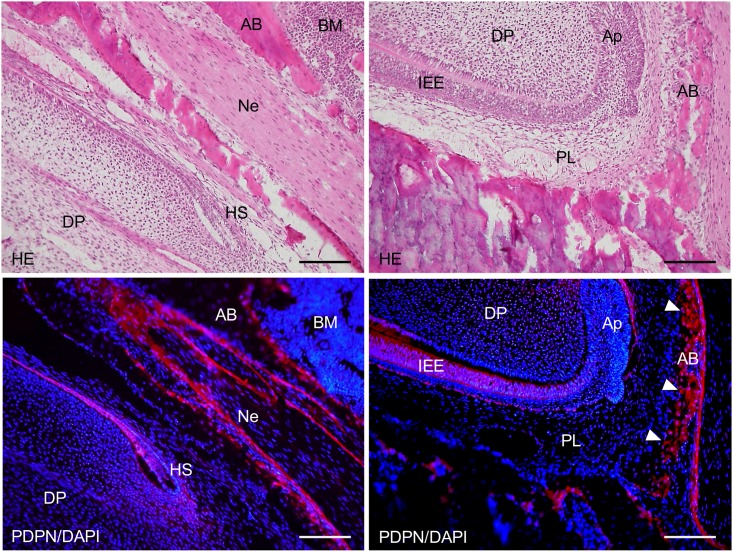
Immunostaining of the 2-week *Wnt1-Cre;Pdpn*^*Δ/+*^ mouse lower incisor sagittal section for podoplanin (PDPN). In hematoxylin-eosin (HE) staining, there are no abnormalities in the tooth germ. In the left images at the higher magnification of the parts highlighted by box (a) in Fig 9, the expression of PDPN is observed in the Hertwig’s epithelial root sheath (HS), in the periosteum at the edge of the alveolar bone (AB), and in the sheath of the nerve (Ne). There is no expression of PDPN observed in the bone marrow (BM) or dental pulp fibroblasts (DP). In the right images at the higher magnification of the parts highlighted by box (b) in Fig 9, the expression of podoplanin is observed in the inner enamel epithelial cells (IEE), in the epithelial cells of the apical bud (Ap), and in the osteocytes of the alveolar bone (AB). There is no expression of PDPN observed in the dental pulp fibroblasts (DP) or in the periodontal ligament (PL). Bar: 100μm.

**Fig 9:**
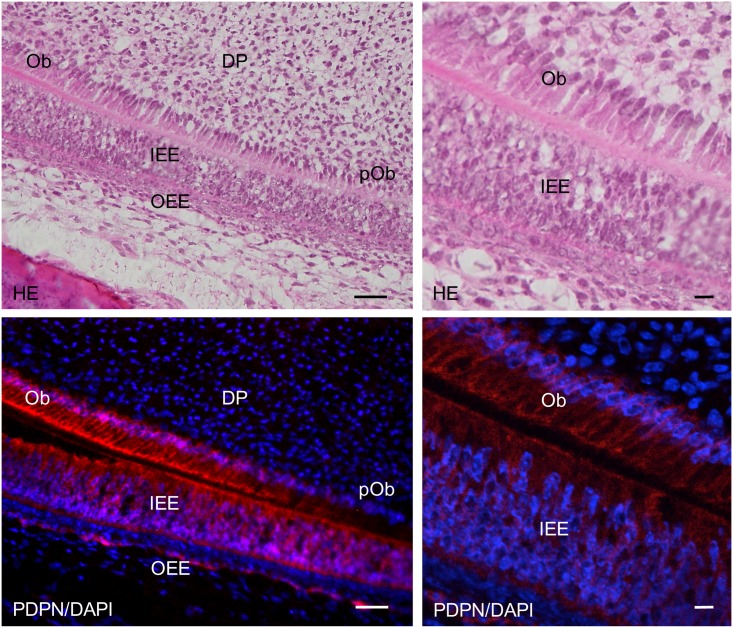
Immunostaining of the 2-week *Wnt1-Cre;Pdpn*^*Δ/+*^ mouse lower incisor sagittal section for podoplanin (PDPN). In the left images at the higher magnification of the parts highlighted by box (c) in Fig 7, the expression of PDPN is observed in the odontoblasts (Ob), in the inner enamel epithelial cells (IEE), and in the outer enamel epithelial cells (OEE), but not in the pre-odontoblasts (pOb). There is no expression of PDPN observed in the dental pulp fibroblasts (DP). In the right images, by confocal microscopy at a higher magnification of the area of the left images, the expression of PDPN is observed on the cell membrane of odontoblasts (Ob) and inner enamel epithelial cells (IEE). Bar: left 100μm, right 10μm.

**Fig 10:**
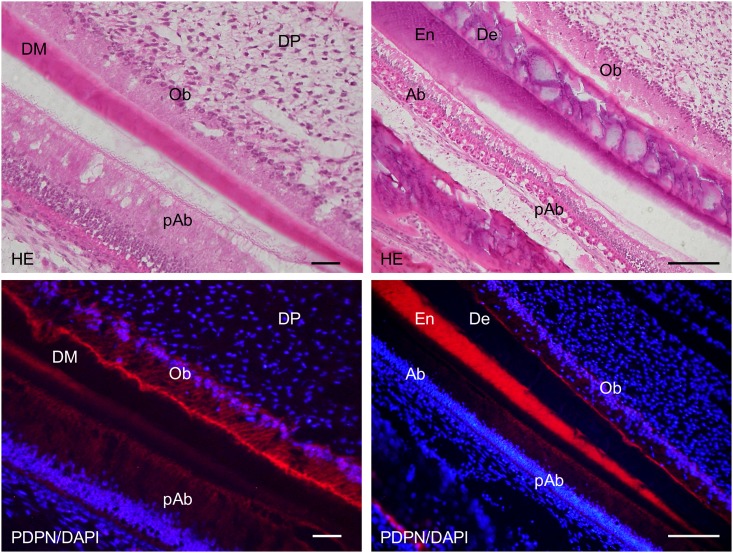
Immunostaining of the 2-week *Wnt1-Cre;Pdpn*^*Δ/+*^ mouse lower incisor sagittal section for podoplanin (PDPN). In the hematoxylin-eosin (HE) staining, there are no abnormalities in the dentin and enamel formation. In the left images at the higher magnification of the parts highlighted by box (d) in Fig 7, the expression of PDPN is observed in the odontoblasts (Ob) forming dentin matrix (DM) and pre-ameloblasts (pAb). The expression of PDPN is not observed in the dental pulp fibroblasts (DP). In the right images at the higher magnification of the parts highlighted by box (e) in Fig 7, the expression of PDPN is observed in the odontoblasts (Ob) and pre-ameloblasts (pAb), with the PDPN expression weaker in the odontoblasts (Ob) where dentin (De) formation has progressed, and weaker in the ameloblasts (Ab) with enamel formation (En). There is cross-reaction to the enamel. Bar: 100μm.

In the 2-week *Wnt1-Cre;Pdpn*^*Δ/+*^ mouse incisors, the expression of podoplanin was observed in the Hertwig’s epithelial root sheath, odontoblasts, apical bud, inner and outer enamel epithelial cells and pre-ameloblasts (Figs 7 and 8). The podoplanin expression was weaker in the odontoblasts where dentin formation progressed (Fig 9) and in the ameloblasts where the enamel formation had started (Fig 10). The podoplanin expression was also observed in the periosteum at the edge of the alveolar bone and the nerve sheath while it was not observed in the dentin, dental pulp fibroblasts, pre-odontoblasts, periodontal ligament, bone marrow, or muscle. There were no abnormalities in the nerve, bone, or tooth development.

**Fig 11:**
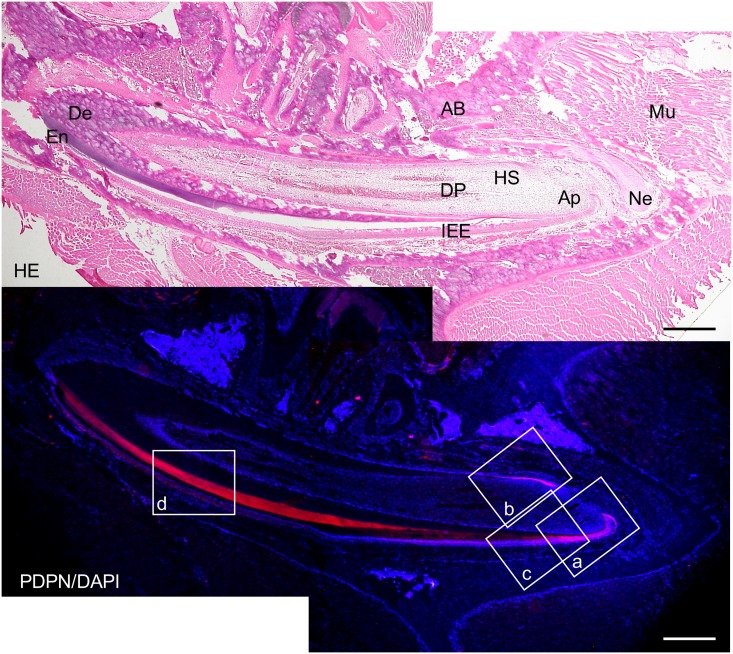
Immunostaining of a 2-week *Wnt1-Cre;Pdpn*^*Δ/Δ*^ mouse lower incisor sagittal section for podoplanin (PDPN). In the hematoxylin-eosin (HE) staining, there are no abnormalities in the bone, dentin, or enamel formation. There is expression of PDPN observed in the epithelial cells: Hertwig’s epithelial root sheath (HS), in the apical bud (Ap), and in the inner enamel epithelial cells (IEE). There is no expression of PDPN observed in the odontoblast layer at the edge of the dental pulp (DP), in the osteocytes of the alveolar bone (AB), or the sheath of the inferior alveolar nerve (Ne). Cross-reaction is observed in the enamel (En) of the labial side but not in the dentin (De), or in the dental pulp fibroblasts (DP), or in the muscle (Mu). Bar: 500μm.

**Fig 12:**
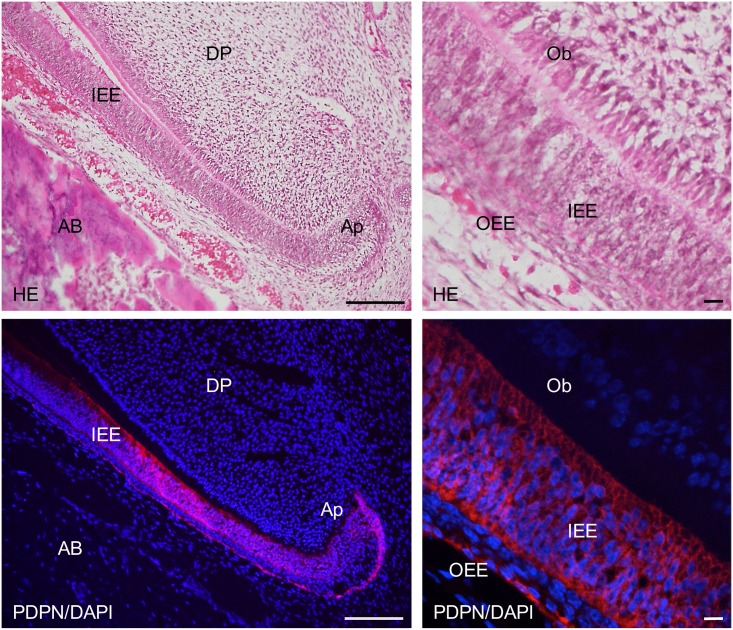
Immunostaining of the 2-week *Wnt1-Cre;Pdpn*^*Δ/Δ*^ mouse lower incisor sagittal section for podoplanin (PDPN). In the left images at higher magnification of the parts highlighted by box (a) in Fig 12, expression of podoplanin is observed in the inner enamel epithelial cells (IEE), and in the epithelial cells of the apical bud (Ap), but not in dental pulp fibroblasts (DP) including odontoblasts, or in the osteocytes of the alveolar bone (AB). In the right images by confocal microscopy at the higher magnification of an area of the left images, expression of PDPN is observed in the inner enamel epithelial cells (IEE) and in the outer enamel epithelial cells (OEE), but not in the odontoblasts (Ob). Bar: left 100μm, right 10μm.

**Fig 13:**
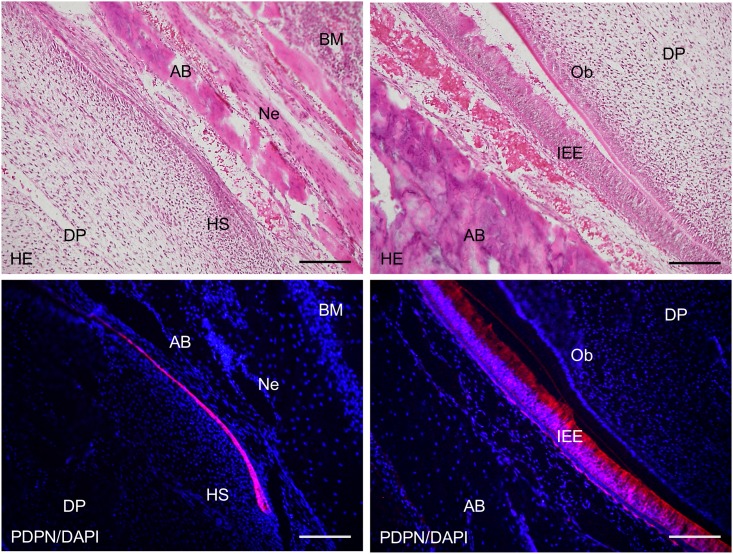
Immunostaining of the 2-week *Wnt1-Cre;Pdpn*^*Δ/Δ*^ mouse lower incisor sagittal section for podoplanin (PDPN). In the left images at a higher magnification of the parts highlighted by box (b) in Fig 11, expression of PDPN is observed in the Hertwig’s epithelial root sheath (HS) but not in the alveolar bone (AB), or in the sheath of the nerve (Ne). There is no expression of PDPN observed in the bone marrow (BM) and dental pulp fibroblasts (DP). In the right images at a higher magnification of the parts highlighted by box (c) in Fig 11, expression of podoplanin is observed in the inner enamel epithelial cells (IEE), but not in the odontoblasts (Ob). There is no expression of PDPN observed in the dental pulp fibroblasts (DP) and in the alveolar bone (AB). Bar, 100μm.

**Fig 14:**
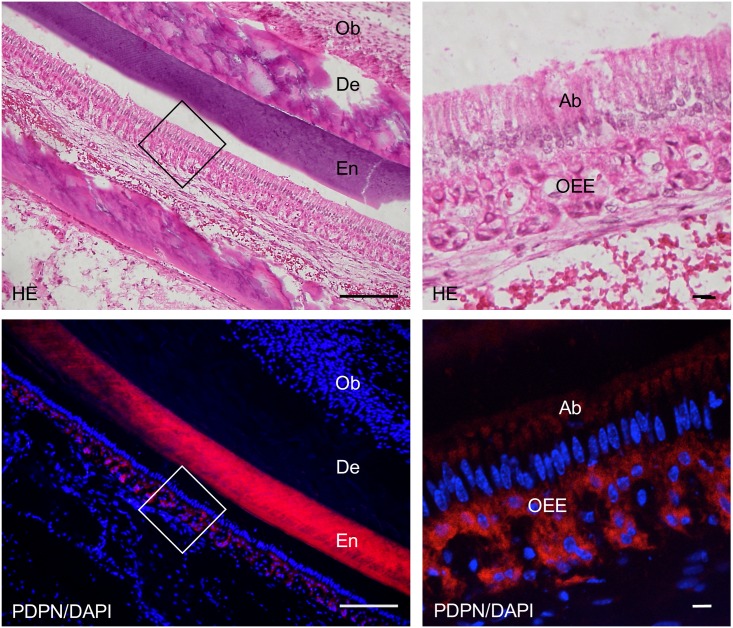
Immunostaining of the 2-week *Wnt1-Cre;Pdpn*^*Δ/Δ*^ mouse lower incisor sagittal section for podoplanin (PDPN). In the hematoxylin-eosin (HE) staining, there are no abnormalities in the dentin and enamel formation. In the left images at the higher magnification of the parts highlighted by the box (d) in Fig 11, PDPN expression is not observed in the odontoblasts (Ob). The immunoreaction is not in the dentin (De) but cross-reaction is observed in the enamel (En). In the right images at a higher magnification of the parts highlighted by box in the left images, podoplanin expression is observed in the outer enamel epithelial cells (OEE), and in the ameloblasts (Ab) with enamel formation to a smaller extent than in the inner enamel epithelial cells without enamel formation before differentiation into the ameloblasts. Bar: left 100μm, right 10μm.

In the 2-week *Wnt1-Cre;Pdpn*^*Δ/Δ*^ mouse lower incisor, the expression of podoplanin was observed in the epithelial cells: Hertwig’s epithelial root sheath, apical bud, inner and outer enamel epithelial cells, and pre-ameloblasts, whereas not observed in the odontoblasts, bone, or nerve sheath, which were PDPN-positive in the *Pdpn*^fl/fl^ (not shown) or *Wnt1-Cre;Pdpn*^*Δ/+*^ mice (Figs 11–14). The expression of PDPN in the ameloblasts where enamel formation had started was weaker than in pre-ameloblasts without enamel formation (Fig 14). There were no abnormalities in the nerve, bone, or tooth development (Figs 11–14).

## Discussion

### Podocyte development in the podoplanin-deficient kidney in the *Pdpn*^KO1st^ mice

Podoplanin has been identified in rat kidney podocyte foot processes [2]. Nephrin is a transmembrane protein that is a structural component of the slit diaphragm. The two are present on the podocytes and maintain the relationship between the basement membrane and podocytes. Nephrin is specifically located at the slit diaphragm of glomerular podocytes. In this study, the expression of podoplanin on podocytes were observed in *Pdpn*^+/-^ mice as well as in the wild type (Fig 2). The expression of nephrin was observed on podocytes and in the area of the diaphragm between podocytes in the wild type, *Pdpn*^+/-^, and *Pdpn*^-/-^ mice, and there were no anomalies in the development of glomeruli in the *Pdpn*^-/-^ mice, suggesting an absence of podoplanin in podocytes causes no morphological disorder in the podocyte processes.

### Alveolus development in the podoplanin-deficient lung of the *Pdpn*^KO1st^ mice

Type I alveolar lung cells express podoplanin [19,20], and the extremely flat and thin type I alveolar cells function as an air-blood barrier. It has been thought that the type II alveolar lung cells differentiate into type I lung cells by podoplanin signaling, and podoplanin expression has been reported in osteocytes and osteoblasts [14–16]. In culture, the podoplanin production is more active, and the amounts are larger in MLO-Y4 osteocyte-like cells than in osteoblasts. Mechanical stress application increases the production of podoplanin and increases the number and lengths of dendrites of the osteocyte-like MLO-Y4 cells. This has led to the hypothesis that podoplanin controls osteocyte morphology and is essential for normal bone function in response to shear stresses. Podoplanin expression has also been reported in mesothelial cells [24,25]. In the thoracic cage and lung in this study, the expression of podoplanin was observed in the pleura mesothelial cells, costal bone, and lung parenchyma in the wild type mice and in the *Pdpn*^+/-^ mice, but not in the *Pdpn*^-/-^ mice (Fig 3). The PDPN expression of the alveoli in the *Pdpn*^+/-^ mice was weaker than in the wild type mice. There were no abnormalities in the development of pleura and costal bone in the *Pdpn*^+/-^ and *Pdpn*^-/-^ mice, but there was atrophy of the lung and disorder of the alveoli observed in the *Pdpn*^-/-^ mice (Fig 3). ImageJ analysis for the immunostaining of lung showed that the expression amounts of podoplanin on lobes and alveoli were significantly higher in the wild type *Pdpn*^+/+^ mice than in the *Pdpn*^+/-^ mice (S1 and S2 Figs). There may be difference between allele products for podoplanin. In the wild type and the *Pdpn*^+/-^ mice, there were PDPN-positive type I alveolar epithelial cells among the TTF-1-positive type II alveolar epithelial cells (Fig 4). The PDPN-positive type I alveolar epithelial cells were fewer in *Pdpn*^+/-^ mice than in the wild type *Pdpn*^+/+^ mice. In the *Pdpn*^-/-^ mice, the alveoli consisted of the alveolar duct with TTF-1-positive type II alveolar epithelial cells but lacked alveolar sacs with type I alveolar epithelial cells. It may be suggested that type II alveolar lung cells with TTF-1 fail to differentiate into type I lung cells in the absence of podoplanin, and that the podoplanin deficient causes no morphological disorder in the development of the pleura and costal bone.

### Tooth germ development in podoplanin-deficient tooth germ in the *Pdpn*^KO1st^ mice

Podoplanin expression has been reported in enamel epithelial cells and odontoblasts in the tooth germ [27,29]. In this study, the expression of podoplanin was observed in the tooth germ, craniofacial bone, nerve sheaths, and Meckel’s cartilage in the wild type mice (Fig 5). In the tooth germ, podoplanin expression was observed in the enamel cord, cervical loop, inner and outer enamel epithelia, and odontoblasts (Fig 5). The PDPN expression in the tooth germ and craniofacial bone was also observed in the *Pdpn*^+/-^ mice to a lesser extent than the wild type mice, but it was not in the *Pdpn*^-/-^ mice (Fig 6). Further, there were no abnormalities in the development of tooth germs, craniofacial bone, and nerves in the *Pdpn*^+/-^ and *Pdpn*^-/-^ mice, suggesting that the podoplanin deficient causes no morphological disorder in the development of tooth germ and craniofacial bone.

### Dentin formation in the *Wnt1-Cre;Pdpn* conditional knockout mouse

The *Wnt1* plays an important role in anterior-posterior patterning in the embryonic central nervous system, and *Wnt1* is also expressed in cranial neural crest-derived craniofacial and odontogenic mesenchymal cells differentiating into odontoblasts, chondrocytes, and osteoblasts [41, 44–46]. Odontogenesis starts with dentinogenesis by the secretion of predentin from odontoblasts following the terminal differentiation controlled by the inner enamel epithelium. Subsequently, the inner enamel epithelium differentiates into ameloblasts by dentin matrix interactions, and ameloblasts secrete enamel matrix. Podoplanin is not expressed in dental pulp cells and pre-odontoblasts, but is expressed in odontoblasts during dentinogenesis, and the podoplanin expression in odontoblasts diminished after dentin formation. Podoplanin is also expressed in the inner and outer enamel epithelical cells, and is lost in the ameloblasts starting the enamel matrix secretion [27,29]. In the 2-week *Wnt1-Cre;Pdpn*^*Δ/+*^ mouse incisors, the expression of PDPN was observed in the oral epithelial cells and oral epithelial cell-derived tooth germ epithelial cells: Hertwig’s epithelial root sheath, apical bud, and inner and outer enamel epithelial cells, and in the periosteum at the edge of the alveolar bone and the nerve sheath, as well as in the wild type mice and *Pdpn*^fl/fl^ (not shown)(Figs 7 and 8). The expression of podoplanin is not observed in the dental pulp cells or in pre-odontoblasts, but it was observed in odontoblasts, and to a smaller extent in the odontoblasts where dentin formation had progressed and this was also the case in the wild type mice (Fig 9). The PDPN expressions was also observed in pre-ameloblasts, but less so in the ameloblasts where enamel formation had started, as well as in the wild type mice and *Pdpn*^fl/fl^, corresponding to the podoplanin expression pattern previously reported (Fig 10)[27,29]. Therefore, it may be assumed that the insertion of a targeting vector and the deletion of the promoter-driven targeting cassette, remaining two loxP sites before and after *Pdpn* exon3, did not affect to the podoplanin production. There was no cross-reaction to the connective tissue and muscle, suggesting that the immunostaining is successful.

In the 2-week *Wnt1-Cre;Pdpn*^*Δ/Δ*^ mice, there was no expression in the odontoblasts, in the osteocytes of the alveolar bone, or mandibular nerve sheaths, which were PDPN-positive in the *Wnt1-Cre;Pdpn*^*Δ/+*^ mice, whereas the expression of PDPN was observed in the oral epithelial cells and oral epithelial cell-derived tooth germ epithelial cells: Hertwig’s epithelial root sheath, apical buds, and inner and outer enamel epithelial cells (Figs 11–14). The neural crest-derived odontoblasts, chondrocytes, and osteoblasts express *Wnt1* [41, 44–46]. Therefore, it may be assumed that the productions of *Pdpn*^fl/fl^ and podoplanin-conditional knockout in the Wnt1 expressing cells were successful. It has been established that podoplanin plays a key role in the elongation of cell processes by the signaling with RhoA family proteins [12,13]. Since podoplanin expression is dependent on odontoblast differentiation, and is reduced in the odontoblasts and ameloblasts as the dentin and enamel formation progresses, we expected that podoplanin could be a contributing factor in the elongation of odontoblast cell processes and dentin formation. However, there were no morphological anomalies in the alveolar bone or tooth in the 2-week *Wnt1-Cre;Pdpn*^*Δ/Δ*^ mice with podoplanin-deficient odontoblasts and osteocytes (Figs 11–14). The expression of podoplanin in the ameloblasts which had started enamel formation was weaker than in the pre-ameloblasts where no enamel formation had taken place, as was the case in the wild type and *Wnt1-Cre;Pdpn*^*Δ/+*^ mice (Fig 14). In the *Wnt1-Cre;Pdpn*^*Δ/Δ*^ adult mice, the tooth and alveolar bone has grown normally. Therefore, it may be suggested that podoplanin expression in odontoblasts is not associated with the dentin and enamel formation, and that podoplanin expression in osteoblasts does not play a critical role in the bone maintenance under usual circumstances without any stress.

## Supporting information

S1 Fig Immunostaining of the respiratory tree terminal ends in the *Pdpn*^KO1st^ mice for podoplanin (PDPN). In the hematoxylin-eosin (HE) staining, the development of intact alveoli (asterisks) is more frequent in the *Pdpn*^+/+^ mice than in the *Pdpn*^+/-^ mice, whereas alveolar sacs are disordered in the *Pdpn*^-/-^ mice. The expressions of podoplanin on the alveoli (arrowheads) and on the mesothelia of diaphragmatic pleura (arrows) are observed in the wild type *Pdpn*^+/+^ mice and the *Pdpn*^+/-^ mice, but not in the *Pdpn*^-/-^ mice. In the *Pdpn*^+/+^ and *Pdpn*^+/-^ mice, the terminal ends of the respiratory tree, pulmonary alveoli, are found in the lung parenchyma and consists of alveolar sacs and alveolar ducts. The PDPN expression of alveoli in the *Pdpn*^+/-^ mice is weaker than in the *Pdpn*^+/+^mice. Bar: 100μm.
(TIF)

S2 Fig ImageJ analysis for the immunostaining of podoplanin of lung lobes and alveoli. The relative expression amounts of podoplanin were expressed by the mean of the ratio (%): podoplanin-positive area in lung lobes (x20, Fig 3) and alveoli (x200, Fig 4) / scanned area. The expression amounts of podoplanin on lung lobes are significantly higher in the wild type *Pdpn*^+/+^ mice than in the *Pdpn*^+/-^ mice. *Significant in ANOVA (P<0.001).
(TIF)
