# Supplementary figures and images for: Morphological study of tooth development in podoplanin-deficient mice
Source: PLoS One. 2017 Feb 21;12(2):e0171912. doi: 10.1371/journal.pone.0171912 (PMC5319687; doi:10.1371/journal.pone.0171912)

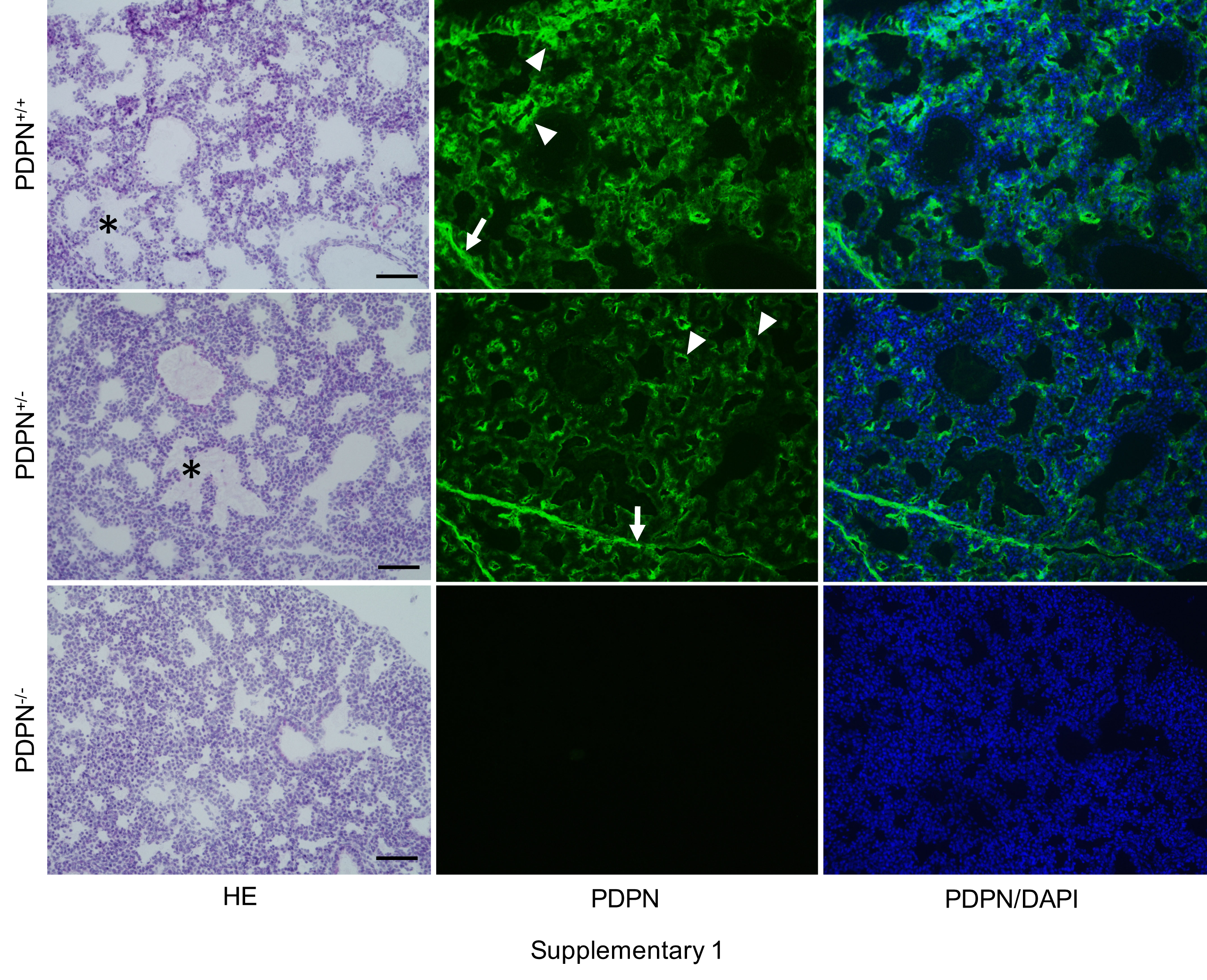

Supplement: S1 Fig — In the hematoxylin-eosin (HE) staining, the development of intact alveoli (asterisks) is more frequent in the Pdpn+/+ mice than in the Pdpn+/- mice, whereas alveolar sacs are disordered in the Pdpn-/- mice. The expressions of podoplanin on the alveoli (arrowheads) and on the mesothelia of diaphragmatic pleura (arrows) are observed in the wild type Pdpn+/+ mice and the Pdpn+/- mice, but not in the Pdpn-/- mice. In the Pdpn+/+ and Pdpn+/- mice, the terminal ends of the respiratory tree, pulmonary alveoli, are found in the lung parenchyma and consists of alveolar sacs and alveolar ducts. The PDPN expression of alveoli in the Pdpn+/- mice is weaker than in the Pdpn+/+mice. Bar: 100μm. (TIF) [file pone.0171912.s001.tif]

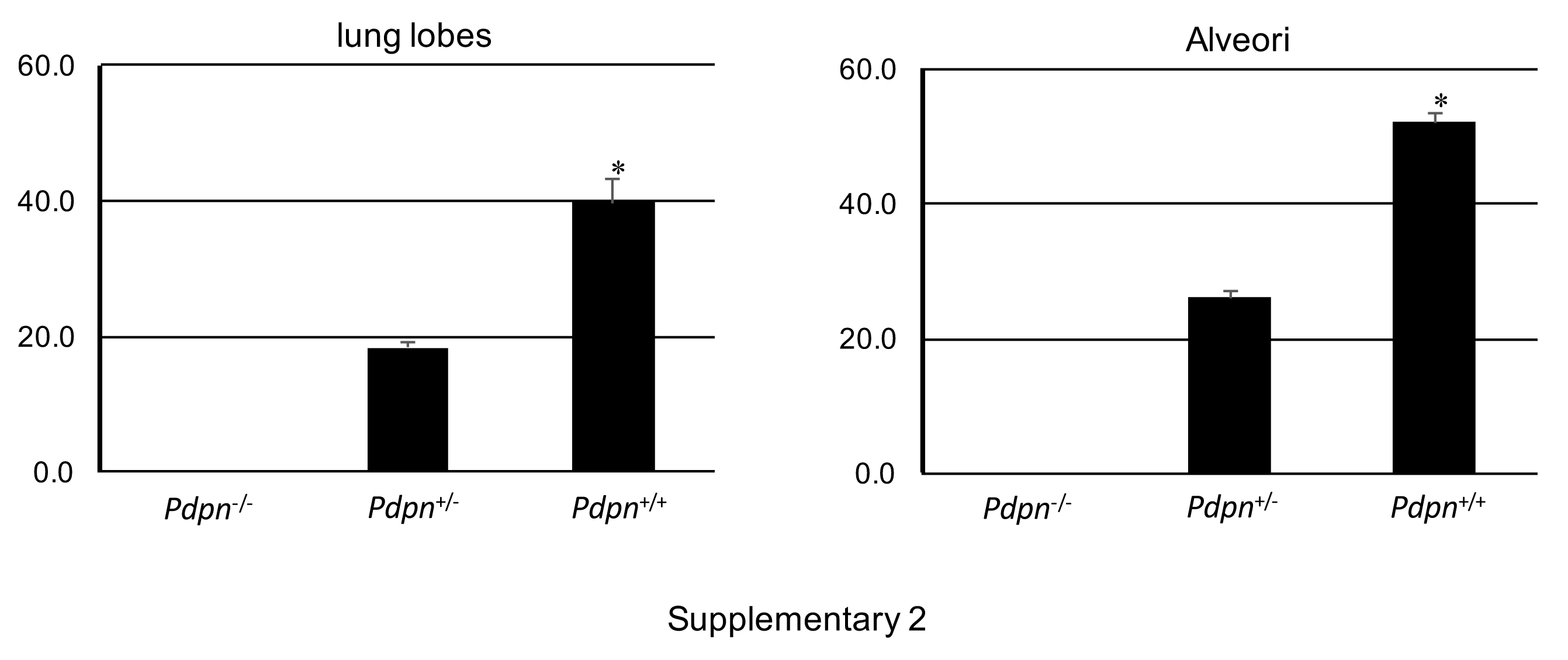

Supplement: S2 Fig — The relative expression amounts of podoplanin were expressed by the mean of the ratio (%): podoplanin-positive area in lung lobes (x20, Fig 3) and alveoli (x200, Fig 4) / scanned area. The expression amounts of podoplanin on lung lobes are significantly higher in the wild type Pdpn+/+ mice than in the Pdpn+/- mice. *Significant in ANOVA (P<0.001). (TIF) [file pone.0171912.s002.tif]
